# Reported Safety Practices of Publicly Advertised Psychedelic Retreats

**DOI:** 10.1001/jamanetworkopen.2025.52505

**Published:** 2026-01-07

**Authors:** Amy L. McGuire, Logan Neitzke-Spruill, Jill O. Robinson, Caroline S. Beit, Nikita Singh, David S. Mathai, Lynnette A. Averill

**Affiliations:** 1 Center for Medical Ethics and Health Policy, Baylor College of Medicine, Houston, Texas; 2 Ethical Legal Implications of Psychedelics in Society Program, Baylor College of Medicine, Houston, Texas; 3 Johns Hopkins School of Medicine, Baltimore, Maryland; 4 Sattva Medicine, Miami, Florida; 5 Department of Psychiatry and Behavioral Sciences, Baylor College of Medicine, Houston, Texas; 6 Michael E. DeBakey Veterans Association, Houston, Texas; 7 Menninger Clinic, Houston, Texas

## Abstract

**Importance:**

The availability of psychedelic retreats has grown to meet the demand for access to these substances. Despite centuries of use among Indigenous communities, psychedelics can pose serious risks for some users.

**Objective:**

To determine safety precautions that retreat organizations that offer psychedelic substances currently use.

**Design, Setting, and Participants:**

This qualitative study included structured interviews conducted by phone or email with representatives from 49 organizations publicly advertising psychedelic retreat offerings from July to October 2023. Organizations were eligible if they marketed their services in English, offered at least 1 psychedelic substance, and made contact information available online. Organizations were selected using convenience sampling from a broader pool of organizations identified in a prior study. Data were analyzed from March 2024 to November 2025.

**Main Outcomes and Measures:**

The main outcomes of interest included the types of drugs offered and presence of polysubstance use, collaboration with health care professionals, presence of health care professionals during retreats, disqualifying conditions, medication washout procedures, and integration practices. Descriptive statistics were used to characterize organizational practices and locations; content analysis was used to categorize medical exclusion criteria, medication washout protocols, involvement of health care professionals, integration offerings, and training of integration facilitators.

**Results:**

Of 48 organizations that reported what substances they offered, all offered either ayahuasca, psilocybin, or both. Nineteen organizations (38.7%) offered more than 1 psychedelic substance. All organizations collected participant medical histories; 36 organizations (73.5%) excluded individuals with certain health conditions. Most (43 organizations [87.8%]) required or recommended medication washout for varying lengths of time, ranging from 1 day to more than 6 weeks. Most (34 organizations [69.4%]) worked with a licensed health care professional or someone with emergency response training, and 32 organizations (65.3%) had someone with those qualifications in attendance at retreats at least some of the time. All organizations offered some sort of integration support.

**Conclusions and Relevance:**

This qualitative study of the practices implemented by psychedelic retreat organizations found substantial variability in the implementation of safety precautions. Some practices related to medication washout and polysubstance use may pose increased risks to participants. Best practice guidelines are needed and should be codeveloped with Indigenous and nonclinical communities.

## Key Points

**Question:**

What safety precautions are implemented by psychedelic retreat organizations?

**Findings:**

This qualitative study with representatives from 49 publicly advertised psychedelic retreat organizations found that most organizations collected medical histories about participants prior to participation, as well as required or recommended cessation of pharmaceutical drugs prior to attendance.

**Meaning:**

These findings suggest that the proliferation of psychedelic retreats can pose safety risks and policy challenges despite the implementation of some safety practices, highlighting the need for continued research and best practice guidance.

## Teaser

This qualitative study describes safety precautions reported by representatives of publicly advertised psychedelic retreat organizations.

## Introduction

Psychedelic policy in the US is rapidly evolving. Department of Health and Human Services (DHHS) Secretary Robert F. Kennedy, Jr., recently endorsed their therapeutic potential, stating that his office aims to make psychedelics available in clinical settings within 12 months despite considerable scientific uncertainty and outstanding questions regarding clinical implementation.^1^ Meanwhile, nonclinical psychedelic use is on the rise, with 8.9% of young adults reporting use in 2023.^2^ In the absence of US Food and Drug Administration approval, users access psychedelics through illicit sources; so-called gray markets, which emerge in circumstances where state or local regulatory changes create legal ambiguities in the absence of official regulatory frameworks; state regulated programs^3^; or international travel to countries with more lenient drug policies and/or religious traditions using psychedelics.^4,5,6^

To meet demand, psychedelic retreat organizations have proliferated, primarily in North and South America, with a considerable number located in the US.^7^ There is considerable variability in the duration and cost of psychedelic retreats, and although only a few publicly advertised retreat organizations purport to be providing medical care,^8^ studies of retreat participants suggest many retreat attendees are seeking relief from serious, often intractable, illnesses, among other motivations.^5,8,9,10^ This trend raises concerns about the potential for physical, psychological, and interpersonal harms stemming from psychedelic use, which are often left for health care practitioners to address without access to medical records or dosing information and with no clear oversight or clinical coordination.

Although psychedelics have been shown to be relatively safe for healthy adults and have been used for centuries in Indigenous traditions, they can pose serious medical and psychiatric risks.^11^ Some psychedelics, like ibogaine, have been shown to be cardiotoxic,^12^ and individuals with cardiac abnormalities may be especially vulnerable to psychedelic use due to sympathomimetic effects and serotonin toxic effects.^13,14^ Those with personal or family histories of psychosis or bipolar disorder also face increased risk of negative effects.^15,16,17^ Furthermore, the psychoactive effects of psychedelics can leave individuals vulnerable to a variety of interpersonal harms. Harm is more likely when retreats lack proper screening, medication oversight, trained medical staff, or adequate integration and follow-up care.^11^

Between 1994 and 2022, 58 participant deaths were reported during ayahuasca retreats.^18^ None of the deaths in events where a cause was determined were attributed to ayahuasca toxic effects and instead were attributed to homicide, suicide, heart attack, drowning, or toxic additions to the ayahuasca brew—indicating that these deaths could have been preventable with proper screening and safety protocols. Yet, little is known about the precautions retreat organizations currently use or how practices vary. Here we report findings from a qualitative study intended to investigate these practices.

## Methods

This qualitative study was approved by the Baylor College of Medicine institutional review board. All participants provided verbal informed consent. The Standards for Reporting Qualitative Research (SRQR) reporting guidelines were followed.

### Study Design and Data Collection

We conducted interviews with representatives who were willing to speak to us from 49 organizations that publicly advertised psychedelic retreat offerings to English-speaking consumers, identified from a broader pool of 298 organizations identified in a prior landscape analysis.^7^ Eligible organizations had online contact information and were selected using convenience sampling; purposive sampling ensured representation outside the US. Up to 3 contact attempts were made. Interviews took place via phone or email between July and October 2023.

Interviews were conducted by a trained research coordinator (C.S.B.), following a structured guide tailored to each organization to confirm and supplement publicly available information (eMethods in Supplement 1). We asked representatives about their organizations’ standard procedures regarding substances offered, safety procedures for screening, medication washout, medical oversight, and the provision and staffing of integration or aftercare services. Responses were recorded via typed notes, entered into REDCap (Vanderbilt) for data management, and exported to Excel 365 (Microsoft) for analysis by 3 team members (L.N.-S., J.O.R., and C.S.B.) Interviews were not recorded because their primary purpose was to verify and supplement data collected online.

### Data Analysis

We used descriptive statistics to characterize organizational practices and locations, categorized as US- or non–US-based. Substances were tallied based on organizational reports. Interview notes were analyzed using content analysis to categorize medical exclusion criteria, medication washout protocols, involvement of health care professionals, integration and aftercare offerings, and the training of integration facilitators.^19^ We used descriptive statistics to characterize the data according to the categories developed through content analysis of interview notes. Data were analyzed from March 2024 to November 2025.

## Results

We contacted 81 eligible organizations; 5 organizations declined and 26 organizations did not respond, yielding a 61.7% response rate. Most interviews were conducted by phone (44 interviews [88.0%]); 6 interviews (12.0%) were conducted by email. One organization was excluded because it did not host retreats. Our final sample included 49 organizations. Most (30 organizations [61.2%]) were US-based. Five organizations (10.2%) self-identified as medical, offering psychedelics for therapeutic purposes or within psychedelic-assisted therapy models (Table 1).

**Table 1.** Characteristics of Retreat Organizations

Characteristic	No. (%) (N = 49)
Organization type^a^	
Medical^b^	5 (10.2)
Religious^c^	27 (55.1)
Wellness^d^	42 (85.7)
Geographic location of organization base	
North America	
Overall	43 (87.8)
Canada	4 (8.2)
Costa Rica	4 (8.2)
Jamaica	2 (4.1)
Mexico	2 (4.1)
Panama	1 (2.0)
US Midwest	2 (4.1)
US Northeast	2 (4.1)
US South	10 (20.4)
US West	16 (32.7)
South America^e^	6 (12.2)
Psychedelic substances offered (n = 48)^a^	
Ayahuasca	30 (62.5)
Psilocybin	28 (58.3)
San Pedro cactus	9 (18.8)
Mebufotenin	6 (12.5)
Dimethyltruptamine	4 (8.3)
Changa	3 (6.3)
Ibogaine	2 (4.2)
>1 Psychedelic	19 (39.6)

^a^ Categories are not mutually exclusive.

^b^ Claimed to use medical techniques or hold medical licensure, used psychedelics for treatment or rehabilitation of specific ailments, or facilitated psychedelic retreats in addition to broader aims of conducting clinical research.

^c^ Required membership to participate, included statements of belief or other religious scriptures, or indicated their religion’s tax-exempt status.

^d^ Portrayed as facilitating personal growth or transformation, self-exploration, healing, or spiritual experiences (absent any other features of religion and within a framework of holistic individual well-being).

^e^ South America included Colombia, Ecuador, Peru.

### Substances Offered

Forty-eight organizations (97.9%) disclosed the psychedelic substances they offered. Ayahuasca (30 organizations) and psilocybin (28 organizations) were most common; all reported offering at least 1 of these. Others included San Pedro cactus (also known as mescaline) (9 organizations), mebufotenin (also known as 5-methoxy-N,N-dimethyltruptamine [DMT]) (6 organizations), DMT (4 organizations), changa (3 organizations), and ibogaine (2 organizations). Two also offered 3,4-methyllenedioxymethamphetamine (MDMA), and 1 offered ketamine. Most (34 organizations [71.4%]) offered more than 1 substance; 19 organizations (38.7%) offered more than 1 psychedelic substance; and 15 organizations (30.6%) offered a psychedelic and a nonpsychedelic substance, like kambo, hapé, cacao, sananga, or cannabis. Of organizations that offered more than 1 psychedelic, some combined multiple psychedelics in a single retreat—eg, ayahuasca on 4 days, San Pedro cactus on 2 days—while others offered different psychedelic substances at separate events.

### Medical Screening Practices

All 49 organizations collected participant medical histories. Thirty-six organizations (73.5%) excluded individuals with certain conditions; 32 organizations (65.3%) specified at least 1 exclusion criterion. Mental health concerns were most common: 26 organizations (53.1%) excluded participants with a mental illness, especially schizophrenia or psychosis (24 organizations [48.9%]) and bipolar disorder (16 organizations [32.7%]). Four organizations also disqualified participants with a family history of psychosis. Cardiovascular and neurological conditions followed in prevalence. A few organizations excluded for other health conditions (Table 2).

**Table 2.** Disqualifying Medical Conditions

Conditions prohibited^a^	No. (%) (N = 49)
Mental health conditions	
Overall	26 (53.1)
Schizophrenia/psychosis	24 (49.0)
Bipolar disorder	16 (32.6)
Personality disorders	6 (12.2)
Substance use disorders	1 (2.0)
Cardiovascular conditions	17 (34.7)
Hypertension	8 (16.3)
Neurological conditions	
Overall	7 (14.3)
Epilepsy or seizures	7 (14.3)
Stroke	4 (8.2)
Endocrine condition, diabetes	6 (12.2)
Pregnancy	6 (12.2)
Other^b^	5 (10.2)
None specified	4 (8.2)

^a^ Categories are not mutually exclusive.

^b^ Eg, AIDS, cancer, eating disorders, irritable bowel syndrome or Crohn disease, asthma or emphysema, recent surgery, history of blood clots.

### Medication Washout Procedures

Thirty-six organizations (73.4%) asked participants about prescription drug use, typically via intake forms or interviews. Seven others did not ask directly but informed participants of medication risks.

Most organizations (43 [87.8%]) required or recommended stopping certain medications before the retreat. Thirty-three organizations (67.3%) mandated discontinuation of drugs, such as selective serotonin reuptake inhibitors [SSRIs], monoamine oxidase inhibitors (MAOIs), lithium, and benzodiazepines. Measures to confirm adherence varied and were not always clear: 1 organization required a drug test; another asked for physician clearance.

Eleven organizations (22.4%) recommended, but did not require, discontinuation of certain medications. One organization required cessation of SSRIs and recommended stopping other medications. Two organizations advised dose reduction on a case-by-case basis. Overall, 31 organizations (63.3%) encouraged participants to consult their physician for tapering guidance; 1 organization deferred entirely to the participant’s own health care practitioner.

Washout timelines varied from 1 day to more than 6 weeks (Table 3). Seven organizations (14.3%) reported consulting medical professionals to guide these protocols.

**Table 3.** Length of Time Required or Recommended for Medication Washout

Washout length of time	Washout, No. (% of total) (N = 49)	
	Recommends (n = 11)	Requires (n = 33)
0 to 7 d	1 (2.0)	2 (4.1)
>1 wk to 2 wk	1 (2.0)	4 (8.2)
>2 to 4 wk	2 (4.1)	11 (22.4)
>4 to 6 wk	NA	4 (8.2)
>6 wk	NA	4 (8.2)
Unspecified amount of time	7 (14.3)	8 (16.3)

### Involvement of Licensed Health Care Professionals

Twenty-one organizations (42.9%) worked with at least 1 licensed health care professional—typically physicians (14 organizations), nurses (12 organizations), therapists (11 organizations), or pharmacists (2 organizations) (Table 4). Their roles included advising on medication washout, screening for contraindications, and supporting participants during or after the retreat. Two organizations noted that these professionals could not legally practice under their licenses in this context. One reported occasional oversight from a non–US-based physician. Of 21 organizations that worked with a licensed health care professional, 6 also worked with emergency-trained personnel (eg, emergency medical technicians or cardiopulmonary resuscitation–certified staff). Among the 28 organizations that did not work with licensed professionals, 13 worked with individuals with emergency response training.

**Table 4.** Information About Access to Health Care Professionals

Type of professional	No. (%) (N = 49)	
	Worked with	In attendance at retreats
Licensed health care professional^a^		
Overall	21 (42.9)	20 (40.8)
Physician	14 (28.6)	9 (18.3)
Nurse	12 (24.5)	13 (26.5)
LCSW or therapist	11 (22.4)	7 (14.3)
Pharmacist	2 (4.1)	1 (2.0)
Other^b^	3 (6.1)	NA
Emergency response personnel only^c^	13 (26.5)	12 (24.5)
Licensed health care professional and emergency response personnel^c^	6 (12.2)	4 (8.2)

Abbreviations: LCSW, licensed clinical social worker; NA, not available.

^a^ Categories are not mutually exclusive.

^b^ Eg, psychologist, physician assistant, or nurse practitioner.

^c^ Emergency response personnel includes firefighter, emergency medical technician, paramedic, or cardiopulmonary resuscitation–certified staff.

Twenty organizations (40.8%) reported that a health care professional was present during at least part of the retreat. Some were available for dosing support, intake assessments, or emergency monitoring. Among organizations without a professional in attendance, 12 relied on emergency-trained staff. In total, 32 organizations (65.3%) had some trained personnel onsite at least occasionally. Two religious groups lacking licensed or emergency-trained staff relied on sober volunteers in case of emergencies.

Among the 5 self-described medical organizations, all worked with licensed professionals. Among the 42 wellness-focused retreats, 17 (40.5%) worked with health care professionals and 16 (38.1%) had them in attendance at retreats. Of the 4 exclusively religious organizations, only 1 reported such involvement.

Twenty-nine organizations (59.2%) said that facilitators used psychedelics during ceremonies, with 7 organizations specifying smaller doses and 4 organizations clarifying that there is always someone sober in attendance. In 3 organizations, licensed professionals also used the substances. Six organizations said facilitator use occurred at their discretion or with participant approval.

### Integration Services

All 49 organizations offered some form of integration support, ranging from structured programs to less structured programs involving informal group circles or ad hoc discussions (Table 5). Thirty organizations (61.2%) included integration activities as part of the retreat program; 18 organizations (36.7%) also offered additional optional services, and 15 organizations (30.6%) only offered optional services. In total, 33 organizations (67.3%) offered optional integration services—7 organizations (14.2%) for an additional fee.

**Table 5.** Types of Integration Activities Offered and Training of Individuals Who Led Integration Activities

Type of integration^a^	No. (%) (N = 49)	
	During retreat	Postretreat
Group sharing circles or group video calls or chat messaging	29 (59.2)	NA
Group video calls or instant messaging	NA	29 (59.2)
1:1 Integration meetings	14 (28.6)	8 (16.3)
Supportive modalities^a^	6 (12.2)	4 (8.2)
Custom self-directed apps, online programs, or books postretreat	NA	8 (16.3)
Recommended writing or journaling	2 (4.1)	3 (6.1)
Integration leader training^b^		
Nonmedical^c^	16 (39.0)	NA
Licensed health care professional^d^	11 (26.8)	NA
Religious^e^	9 (22.0)	NA

Abbreviation: NA, not available.

^a^ Eg, yoga or breathwork.

^b^ Among organizations that disclosed training (n = 41). Categories are not mutually exclusive.

^c^ Eg, life coach, certified integration specialist, certified psychedelic practitioner.

^d^ Eg, therapy (licensed clinical social worker or licensed marriage and family therapist, psychologist), psychiatry, nursing.

^e^ Eg, shamanic or other religious training.

Forty-one organizations (83.7%) disclosed the training of integration facilitators. Most were not licensed professionals but had nonmedical or spiritual credentials, such as life coaches, certified integration specialists, or shamans (Table 5).

In addition to integration, 15 organizations (30.6%) offered structured preparation activities. These included intention setting, group meetings to introduce facilitators and participants, or individual preparatory calls.

## Discussion

This qualitative study with representatives from psychedelic retreat organizations provides new insights into how psychedelic retreats mitigate—and sometimes exacerbate—medical risks. As interest in psychedelics grows, retreats will be increasingly sought for healing, spiritual exploration, and personal growth. Yet the safety protocols guiding these experiences remain inconsistent and poorly documented. Our findings highlight wide variability in screening, medication management practices, and integration services, offering a foundation for future best practices and consumer guidance. Of course, it is vital to note that conceptions of what constitutes safe practice from a Western medical perspective may vary from accepted norms in Indigenous traditions, religious practices, or neo-shamanic contexts.

All organizations used at least some safety measures, most commonly screening for contraindicated medical or psychiatric conditions. Schizophrenia and bipolar disorder were the most frequent disqualifiers. However, screening protocols varied widely, and all relied on self-report, which may be inaccurate—especially among individuals desperate for relief from treatment-resistant conditions, like posttraumatic stress disorder, depression, or addiction.^8,20,21,22,23,24^

Integration services were universally offered but inconsistent in depth and formality. Most included optional activities, like sharing circles, often led by nonmedical personnel. Structured integration—a hallmark of clinical psychedelic therapy—was the exception, which suggests there may be opportunities for improved support for participants returning home. Yet, even in clinical research, there are still significant outstanding questions surrounding the necessity and extent of integration services required. Many participants in psychedelic retreats may not be experiencing clinical symptoms and thus may not require the level of structured integration offered in clinical research settings. Nevertheless, beyond the aims of harm-reduction, more structured integration services may serve as a form of benefit-maximization even for healthy participants.^25^

Some practices could increase risk of adverse outcomes. Nearly 90% of organizations required or recommended medication washout, often for drugs like SSRIs or MAOIs. This is likely driven by concerns about serotonin syndrome and dulling of psychedelics’ psychoactive effects. However, evidence suggests serotonin syndrome is rare,^26,27,28,29^ although it varies depending on which substances are involved.^29,30^ Still, medication washout procedures may stem in part from the desire to ensure participant satisfaction by removing potential hindrances to psychedelics’ vivid subjective effects. While it has been shown that psilocybin, for example, remains effective for depression even with concurrent SSRI use,^31,32^ some evidence suggests that the subjective experience of psychedelics may be impacted by SSRIs and other antidepressants.^26,27,28,33^ Nonetheless, medication washout procedures may exacerbate medical risks or psychological vulnerability, since abrupt discontinuation of antidepressants carries known withdrawal risks and potential for symptom relapse.^34,35^ Given the variable washout timelines—ranging from 1 day to more than 6 weeks—further research and clearer guidance are needed.

Concerns also arise around polysubstance use. Nine organizations reported offering multiple psychedelics within a single retreat. The safety implications of combining substances like ayahuasca, San Pedro cactus, and mebufotenin in close succession remain understudied. Preliminary evidence suggests polysubstance use may increase adverse psychological effects.^36^

Most organizations (32 organizations [65.3%]) had some form of trained personnel in attendance at retreats, including health care professionals or individuals with emergency response training. However, roles and responsibilities were often vague. Some professionals reportedly consumed psychedelics alongside participants, potentially compromising their capacity to respond to emergencies in cases where there are no other sober attendants. Yet, the issue of facilitator coconsumption is a notable point of divergence between clinical and traditional or community perspectives. For example, it is common and even considered necessary in some Indigenous traditions, such as Peruvian *Vegetalismo,* for practitioners to use psychedelics in training and to consume psychedelic medicines, often at smaller doses, as part of their ceremonial healing practices.^25,37,38,39^

In international settings, licensed professionals may not be authorized to practice under their credentials, and few organizations acknowledged this. In most cases, the nature of these professionals’ relationships with the retreat—contractual, consultative, or informal—was unclear. These ambiguities complicate both participant safety and professional accountability. While greater transparency is needed around the qualifications and roles of health care professionals involved in psychedelic retreats, overemphasis on professional credentials as a marker of expertise may obscure the importance of other types of experts, such as Indigenous shamans or peer support specialists, who may leverage knowledge gained from years of experience working with psychedelics to reduce harm.

### Limitations

This study has several limitations. First, it reflects a Western medical framework, which may not align with Indigenous or non-Western conceptions of health and safety. Many retreats draw from ceremonial traditions with distinct cultural logic. Imposing Western standards risks cultural insensitivity; future best practices should be codeveloped with Indigenous and nonclinical communities.

Second, our sampling was limited to organizations with publicly available English-language contact information. As a result, US-based, medically oriented, and commercial retreats may be overrepresented. For instance, commercial retreat organizations may be more apt to take safety risks, such as hosting participants exhibiting risky contraindications, to increase revenue. In addition, nonresponders may differ in important ways. However, the diversity observed in this study suggests our findings still capture key trends.

Third, the retreat landscape is rapidly evolving. Practices may shift in response to legal, social, or market pressures. Our data offer a snapshot in time. Additionally, all data were self-reported, and organizations may have underrepresented or overrepresented their practices, especially given the legal ambiguity of psychedelic use in many jurisdictions. Reliance on written notes to record interview responses may have led to researcher-introduced bias regarding what was recorded or omitted, despite attempts to be comprehensive.

Furthermore, our study does not evaluate the relationship between specific types of safety practices and the rates of adverse outcomes from attending psychedelic retreats. Further research should explore what safety practices are warranted or necessary for minimizing adverse events, particularly for participants who may be seeking treatment for a medical condition through participation in a psychedelic retreat.

## Conclusions

In this qualitative study of psychedelic retreat organizations, we found that organizations implemented a variety of practices to screen, manage medications, and facilitate integration for participants in psychedelic retreats that may variously maximize safety or pose risks. As interest in psychedelic retreats accelerates, so does the need for transparency, safety standards, and informed decision-making. While many retreat organizations implement thoughtful safety practices, substantial variability and lack of oversight remain. Consumers and health care professionals must navigate this landscape with caution. Our findings underscore the need for clearer guidance. While professional organizations, such as the American Medical Association, American Nurses Association, and American Psychological Association, can help licensed clinicians navigate their professional responsibilities, there is also a need for ethical standards that are cocreated among relevant stakeholders, including researchers, clinicians, retreat organizers and participants, representatives from Indigenous communities, and leaders from existing peer support networks. Further research is needed to reduce harm and support positive outcomes in nonclinical psychedelic settings.

## Notes

Supplement 1.
eMethods.

Supplement 2.
Data Sharing Statement
