# Supplementary material for: Reported Safety Practices of Publicly Advertised Psychedelic Retreats
Source: JAMA Netw Open. 2026 Jan 7;9(1):e2552505. doi: 10.1001/jamanetworkopen.2025.52505 (PMC12780929; doi:10.1001/jamanetworkopen.2025.52505)
Supplement: Supplement 1. — eMethods. [file jamanetwopen-e2552505-s001.pdf]

Supplemental Online Content

McGuire AL, Neitzke-Spruill L, Robinson JO, et al. Reported safety practices of publicly advertised psychedelic retreats. *JAMA Netw Open*. 2026;9(1):e2552505.
doi:10.1001/jamanetworkopen.2025.52505

eMethods.

This supplemental material has been provided by the authors to give readers additional information about their work.

© 2026 McGuire AL et al. *JAMA Network Open*.

eMethods

Retreat Organization Practices Data Collection Template

Retreat Name:
Phone:
Email:
Contact Us Form:

Script:
Hi. My name is [Name]. I am a research coordinator working at Baylor College of Medicine. We are very interested in psychedelic retreats and are researching what is currently being offered. We have collected some information online but would like to confirm that the information we have is correct. We are looking at this data across a number of retreats and won't be reporting any individual information so what you tell us will remain confidential. Would you be willing to confirm a few things for me?

1. *Where are you headquartered or based?*
2. *Where do your retreats take place?*
3. *Who typically attends your retreats and how do they find out about your organization?*
4. *It says on your website that you offer [substance(s)…] retreats. Is that correct? Do you offer any other types of psychedelic retreats?*
5. *Can you confirm for me what happens on the retreat?*
    a. *How much [substance(s)…] do participants take? Is it just one session or multiple sessions?*
    b. *How long does it last (how long is the retreat, how long is each session)?*
    c. *How much does it cost?*
6. *Are there facilitators attending the retreats?*
    a. *How are the facilitators trained and found?*
    b. *Do the facilitators also take the [substance(s)?]*
7. *Do you have medical professionals on staff?*
    a. *If yes: What kind of professional? In what capacity (what is their role)?*
8. *Do you have medical professionals attending the retreats?*
    a. *If yes: What kind of professional? In what capacity (what is their role)?*
9. *What do you suggest or require people do if they are currently taking psychiatric medications? [Ask confirmation questions about medical screening here]*
10. *What kind of integration do you offer (during the retreat and after the retreat has ended)? Who leads it? Is it required?*

© 2026 McGuire AL et al. *JAMA Network Open*.
