# Supplementary material for: Reported Safety Practices of Publicly Advertised Psychedelic Retreats
Source: JAMA Netw Open. 2026 Jan 7;9(1):e2552505. doi: 10.1001/jamanetworkopen.2025.52505 (PMC12780929; doi:10.1001/jamanetworkopen.2025.52505)
Supplement: Supplement 2. — Data Sharing Statement [file jamanetwopen-e2552505-s002.pdf]

Data Sharing Statement

McGuire. Reported Safety Practices of Publicly Advertised Psychedelic Retreats. *JAMA Netw Open*. Published January 07, 2026. doi:10.1001/jamanetworkopen.2025.52505

Data
Data available: No

Additional Information
Explanation for why data not available: Individual level note templates contain identifiable information and cannot be made available to others.
